# Intravital imaging of hemodynamic glomerular effects of enalapril or/and empagliflozin in STZ-diabetic mice

**DOI:** 10.3389/fphys.2022.982722

**Published:** 2022-09-12

**Authors:** Hannah Kroeger, Friederike Kessel, Jan Sradnick, Vladimir Todorov, Florian Gembardt, Christian Hugo

**Affiliations:** Experimental Nephrology and Division of Nephrology, Department of internal Medicine III, University Hospital Carl Gustav Carus at the Technische Universität Dresden, Dresden, Germany

**Keywords:** SGLT2 inhbition, ACE inhibition, single nephron GFR, hemodynamic, intravital 2-photon microscopy, Type 1 diabetes mellitus, STZ (streptozocin)

## Abstract

**Background:** Diabetic kidney disease is the leading cause of end-stage renal disease. Administration of ACE inhibitors or/and SGLT2 inhibitors show renoprotective effects in diabetic and other kidney diseases. The underlying renoprotective mechanisms of SGLT2 inhibition, especially in combination with ACE inhibition, are incompletely understood. We used longitudinal intravital microscopy to directly elucidate glomerular hemodynamics on a single nephron level in response to the ACE inhibitor enalapril or/and the SGLT2 inhibitor empagliflozin.

**Methods:** Five weeks after the induction of diabetes by streptozotocin, male C57BL/6 mice were treated with enalapril, empagliflozin, enalapril/empagliflozin or placebo for 3 days. To identify hemodynamic regulation mechanisms, longitudinal intravital multiphoton microscopy was employed to measure single nephron glomerular filtration rate (snGFR) and afferent/efferent arteriole width.

**Results:** Diabetic mice presented a significant hyperfiltration. Compared to placebo treatment, snGFR was reduced in response to enalapril, empagliflozin, or enalapril/empagliflozin administration under diabetic conditions. While enalapril treatment caused significant dilation of the efferent arteriole (12.55 ± 1.46 µm vs. control 11.92 ± 1.04 µm, *p* < 0.05), empagliflozin led to a decreased afferent arteriole diameter (11.19 ± 2.55 µm vs. control 12.35 ± 1.32 µm, *p* < 0.05) in diabetic mice. Unexpectedly under diabetic conditions, the combined treatment with enalapril/empagliflozin had no effects on both afferent and efferent arteriole diameter change.

**Conclusion:** SGLT2 inhibition, besides ACE inhibition, is an essential hemodynamic regulator of glomerular filtration during diabetes mellitus. Nevertheless, additional mechanisms—independent from hemodynamic regulation—are involved in the nephroprotective effects especially of the combination therapy and should be further explored in future studies.

## Introduction

Diabetes mellitus is the main reason for end-stage renal disease (ESRD) in the Western world (Kovesdy, 2022). The number of patients with ESRD is expected to double in the next 10-15 years further increasing treatment costs. Despite tremendous efforts and ongoing research, until recently there was no specific therapy for diabetic nephropathy (DN) or other chronic progressive kidney diseases (CKD) besides angiotensin-converting enzyme (ACE) inhibition (Joannidis and Hoste, 2018; Kovesdy, 2022). One characteristic of early stage changes of DN is the activation of the renin-angiotensin system (RAS), resulting in vasoconstriction of the efferent arteriole and increasing intraglomerular pressure (Giani et al., 2021). RAS blockade with angiotensin-converting enzyme inhibitors (ACEi) or angiotensin-receptor blockers (ARBs) was the gold standard to slow down the development and progression of any CKD, including DN (Joannidis and Hoste, 2018).

Sodium-glucose cotransporter 2 (SGLT2) inhibitors are a new, recently approved therapy for diabetes mellitus type 2 (Wanner et al., 2016), also with beneficial effects in type 1 diabetes (Henry et al., 2015). SGLT2 inhibitors (SGLT2i) are the first class of antidiabetic drugs directly acting on the kidney and exhibiting nephroprotective effects (Zinman et al., 2015; Wanner et al., 2016). Additionally, recent clinical trials in different cardiovascular/diabetic high risk patient groups have demonstrated that SGLT2i such as empagliflozin, dapagliflozin, or canagliflozin reduce the risk for death, as well as cardiac and renal outcomes (Zinman et al., 2015; Wanner et al., 2016; Neal et al., 2017; Wiviott et al., 2019).

The tubuloglomerular feedback (TGF) plays an important role in maintaining intraglomerular pressure and glomerular filtration rate (GFR) (Vallon, 2003). TGF is a negative feedback loop at the juxtaglomerular apparatus that stabilizes GFR and distal salt delivery at the *macula densa* (Vallon, 2003; Schnermann and Castrop, 2013). Under diabetic conditions, high amounts of glucose are reabsorbed by SGLT2 in the early proximal tubule. The subsequent increase in Na^+^-reabsorption lowers its distal delivery at the *macula densa*. Consequently, activation of TGF leads to vasodilation of the afferent arteriole, thus increasing intraglomerular pressure and GFR (Heerspink et al., 2016). Considering that SGLT2 is solely expressed within the proximal tubules (Chen et al., 2010), overall- and nephroprotective effects in humans are likely to be mediated via the kidneys (Zinman et al., 2015; Wanner et al., 2016). Furthermore, recent results demonstrated that the concurrent administration of ACEi and SGLT2i improves renal outcomes synergistically (Kojima et al., 2015; Wanner et al., 2016). The underlying mechanisms, linking arteriole width alterations, intraglomerular pressure regulation, and sodium delivery to the *macula densa* - subsequently slowing down the progression of CKD - are not fully understood. SGLT2i alone may normalize GFR under diabetic conditions by restoring the TGF mechanism (Nespoux and Vallon, 2018). Consistent with this hypothesis, we and others have shown that SGLT2 inhibition reduced renal and glomerular hypertrophy and kidney injury in experimental diabetic nephropathy (Gembardt et al., 2014).

Based on the modes of action, simultaneous ACE and SGLT2 inhibition may have synergistic effects regarding restoration of TGF under diabetic conditions. While synergistic hemodynamic effects by combination therapy of nonsteroidal anti-inflammatory drugs (NSAIDs) and ACEi/ARBs are known to frequently cause severe tubular necrosis and compromised renal function via glomerular hypotension (Seelig, Maloley and Campbell, 1990), it remains unclear why SGLT2i together with ACEi/ARBs prevents from acute renal failure and are especially effective in reno- and cardioprotection (Kojima et al., 2015; Zhang et al., 2018; Sridhar, Tuttle and Cherney, 2020).

Therefore, our experimental study for the first time investigates glomerular hemodynamic regulation of ACE inhibition with enalapril or/and SGLT2 inhibition with empagliflozin at the single nephron level in mice *in vivo*. Hereby, we used sophisticated intravital multiphoton imaging techniques to directly visualize glomerular hemodynamics by longitudinal, repetitive assessment of single nephron GFR (snGFR) and afferent as well as efferent arteriole width in STZ-induced type 1 diabetic mice at baseline conditions and in response to ACEi or/and SGLT2i within the same animals.

## Material and methods

### Animals

Diabetes was induced in male C57BL/6 (Janvier) mice at 6-7 weeks of age by intraperitoneal injections of streptozotocin (STZ; Sigma-Aldrich) in sodium citrate buffer (pH 4.5) at a dose of 50 mg/kg for five consecutive days. Non-diabetic controls received equal amounts of buffer alone. Blood glucose levels were determined 1 week after the last injection of STZ. Mice with blood glucose level above 20 mmol/l were considered as diabetic. Healthy controls and STZ-diabetic mice were randomly divided into four groups (1. Control; 2. 50 mg/l enalapril in drinking water; 3. 300 mg/kg empagliflozin in chow; 4. co-treatment with enalapril and empagliflozin; 50 mg/l enalapril and 300 mg/kg empagliflozin) 5 weeks later. The control group (placebo) received standard chow and drinking water without any drugs.

For urine collection, mice were housed individually in metabolic cages for 24 h. The mice had free access to food and water in the metabolic cages. Blood samples were obtained from non-fasting animals after 16 days of treatment. Blood samples were centrifuged for 15 min at 2.500 × *g*, and the collected serum was stored at -20°C for further analysis. Urinary and serum samples were analyzed at the Clinical Chemistry at the University Hospital Carl Gustav Carus (Dresden, Germany) using standard laboratory methods to determine different parameters such as serum glucose, serum ACE activity, and urinary glucose.

Transdermal measurement of glomerular filtration rate (GFR) was performed as described previously using FITC-sinistrin (Schreiber et al., 2012).

All animal experiments were performed in accordance with the Federal Law on the Use of Experimental Animals in Germany and were approved by local authorities (Az. 25-5131/496/48 and Az. 24-9168.11/1/380).

### Intravital multiphoton microscopy

For intravital microscopy, only STZ-diabetic mice were used. First, the mice were anesthetized with isoflurane (0.8 l/min, 2.5%, Baxter Deutschland GmbH), and an abdominal body window was implanted for repeated kidney imaging as previously described (Schiessl et al., 2019).

The next day, mice were anesthetized with isoflurane (0.8 l/min, 1.5%), intubated, and catheterized into the lateral tail vein. To maintain body temperature during intravital microscopy, the mice were kept on a heating plate.

Image acquisition was performed using an upright Leica SP8 multiphoton laser scanning microscope with a 40x/1.1 NA water immersion objective at the Core Facility Cellular Imaging at the Technical University Dresden. Multiphoton imaging was performed with 860 nm laser excitation to visualize Angiospark680® (Perkin&Elmer, 30 µl), Hoechst 33,342 (Thermo Fischer, 50 µl of 2 mg/ml stock), and Lucifer Yellow (LY; Sigma-Aldrich).

### Single nephron GFR measurement

snGFR measurement in living mice was measured as previously described (Kang et al., 2006). In brief, superficial glomeruli with a subsequent proximal tubule (PT; minimum 45 µm length) were used for analysis. An automated syringe pump injected the freely filtering LY (15 µl of 5 mg/ml stock) into the lateral tail vein. A time series (6 frames/s) was acquired during the application of LY and was used to calculate snGFR as we previously described (Kessel et al., 2021).In short, semi-automatic image analysis was programmed using FIJI (Schindelin et al., 2012) and data analysis using R (R Core Team, 2017) with RStudio (RStudio Team, 2019). For image analysis, the position and direction of the flow along the proximal tubule had to be manually set. Afterwards, LY intensity was measured over time in every frame automatically. The selection further provides PT length and mean PT diameter to calculate PT volume. Finally, snGFR is automatically calculated as volume change over time. Number of animals/number of snGFR measurements: *n* (Placebo) = 8/13, *n* (Enalapril) = 5/9, n (Empagliflozin) = 5/9, *n* (Enalapril/Empagliflozin) = 5/7. The number of measurement is limited to three nephrons per animal.

### Measurement of arteriole diameter and glomerular volume

For measurement of afferent and efferent arteriole diameter in living mice, a z-stack (1 µm z-size over 120 µm length) of superficial glomeruli was captured. The repeated measurement of arteriole diameter was performed without a cortical slice as mentioned in other experimental setups (Satoh et al., 2010; Kidokoro et al., 2019). Further analysis was performed in Imaris (version 9.5.0, Bitplane). The afferent and efferent arteriole were identified by the direction of blood flow. Afterwards, the arterioles were marked in every plane of the z-stack, three-dimensionally reconstructed, and the mean diameter was calculated automatically. For glomerular volume, the glomerulus was three-dimensional reconstructed by identification of the glomerular capillaries in every plane of the z-stack. Afterwards, the glomerular volume was automatically calculated. Number of animals/number of afferent arteriole measurements: *n* (animals/AA): *n* (Placebo) = 5/11, *n* (Enalapril) = 2/5, *n* (Empagliflozin) = 5/7, *n* (Enalapril/Empagliflozin) = 8/14. Number of animals/number of efferent arteriole measurements: n (animals/EA): *n* (Placebo) = 4/7, *n* (Enalapril) = 2/4, *n* (Empagliflozin) = 4/6, *n* (Enalapril/Empagliflozin) = 6/14. The number of animals for arteriole width measurements varies between the individual groups, because depending on the orientation of the glomeruli to the microscope objective, the measurement of the afferent and efferent arteriole diameter is not possible in all glomeruli at each time point.

### Statistical analysis

Data are shown as dot plot ± standard deviation (SD). The data were visualized and analyzed using R (version 4.0.2.) (R Core Team, 2017) with RStudio (version 1.2.5033) (RStudio Team, 2019). Comparison between multiple groups was performed by using one-way ANOVA followed by the Šídák’s multiple comparison test. Comparison between two groups was performed using an unpaired or paired 2-tailed Student t-test. *p* values < 0.05 were considered statistically significant.

## Results

### Basic parameters

First, we analyzed the clinical changes in non-diabetic control and STZ-diabetic mice. Before the mice were randomly divided into treatment groups, diabetic mice had a significantly lower body weight (Table 1), higher serum glucose levels (data not shown), and higher amounts of urinary glucose (Table 1) than the non-diabetic controls. Treatment with enalapril, empagliflozin, and enalapril/empagliflozin itself had no impact on body weight or kidney weight. However, diabetic mice had significantly higher kidney weights than the controls without an impact of the different treatments (Table 1).

**TABLE 1:** Basic parameters of non-diabetic control mice and STZ-diabetic mice. Values are shown before mice were randomly divided into groups (at randomisation) and after treatment with placebo, enalapril, empagliflozin, and enalapril/empagliflozin. BW indicates bodyweight, KW/BW, kidney weight to body weight ratio, s-Glucose, serum-Glucose, ACE-Angiotensin Converting Enzyme, and u-Glucose, urinary Glucose, nd, not detected. At randomisation an unpaired *t*-test was performed for statistical difference. For comparison between the treatment groups an one-way ANOVA was performed, *n* (non-diabetic controls) = 10-22, except for ACE activity in the enalapril group (*n* = 3), *n* (diabetic) = 5-16. All values are expressed as mean ± SD.

	Non-diabetic control					Diabetic				
Group	At randomi-sation	Placebo	Enalapril	Empagliflozin	Enalapril/Empagliflozin	At randomi-sation	Placebo	Enalapril	Empagliflozin	Enalapril/Empagliflozin
BW [g]	25.5 ± 2.0	26.4 ± 2.5	26.1 ± 1.3	25.0 ± 1.5	24.1 ± 2.3	21.6 ± 2.6§	20.9 ± 2.2	21.2 ± 2.1	20.4 ± 1.1	22.5 ± 2.6
KW/BW [mg/g]	-	6.5 ± 1.1	5.8 ± 0.7	6.9 ± 0.8	6.3 ± 1.0	-	8.2 ± 1.0*	7.1 ± 0.7	8.1 ± 0.9	7.9 ± 1.0
u-Glucose [mmol/24 h]	nd	nd	nd	1.8 ± 0.6	1.9 ± 0.5	6.6 ± 3.4§	8.0 ± 3.8	5.9 ± 1.9	7.0 ± 2.7	4.3 ± 1.7
ACE [U/L]	-	404.5 ± 71.7	117.7 ± 27.7*	409.8 ± 50.3	71.1 ± 28.2*	-	437.6 ± 52.4	74.7 ± 34.6#	415.3 ± 54.1	55.4 ± 32.2#
Urine volume [ml/24 h]	1.0 ± 0.6	0.9 ± 0.4	1.1 ± 0.4	2.0 ± 0.9	2.1 ± 1.3	11.2 ± 5.2§	13.8 ± 5.8	13.5 ± 3.8	11.9 ± 6.8	7.1 ± 4.0

*p < 0.05 vs. non-diabetic control-placebo, #*p* < 0.05 vs. diabetic-placebo, §*p* < 0.05 vs. non-diabetic control-at randomisation.

**FIGURE 1:**
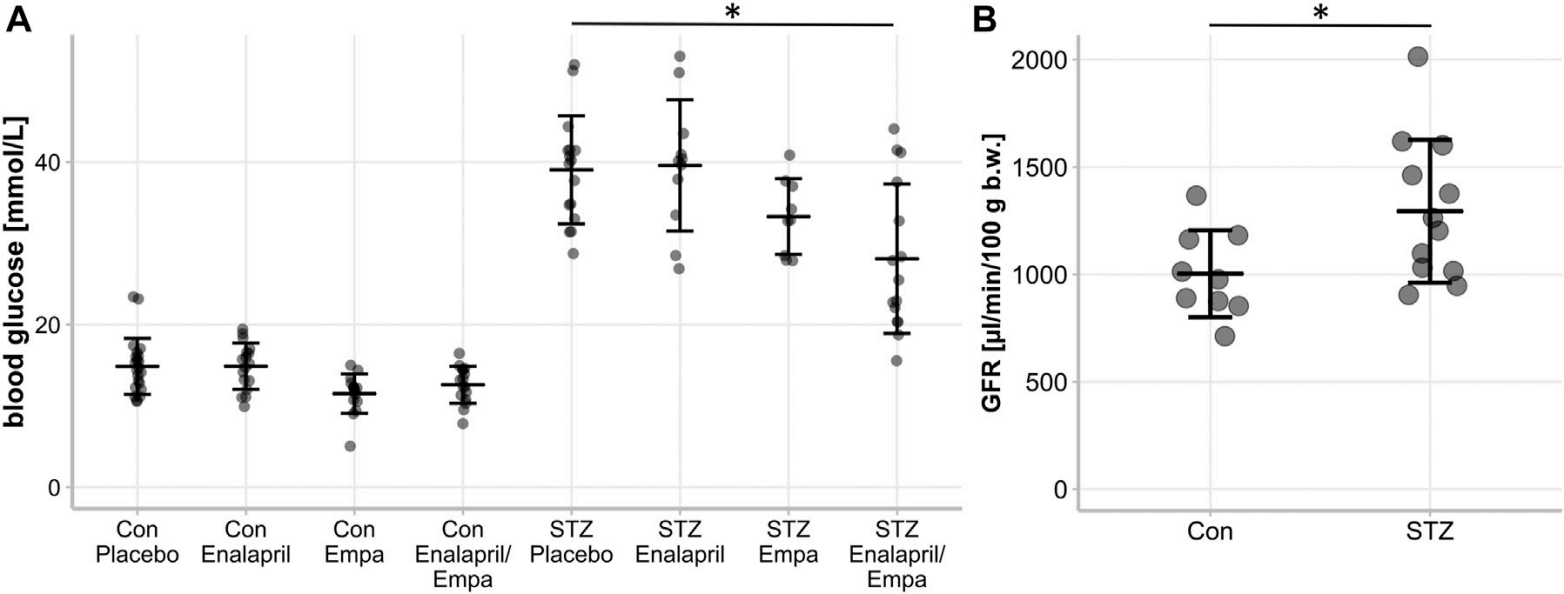
Blood glucose level and glomerular filtration rate (GFR) of non-diabetic control mice and STZ-diabetic mice. Effect of enalapril, empagliflozin (Empa), and enalapril/empagliflozin (Enalapril/Empa) treatment on **(A)** blood glucose and **(B)** glomerular filtration rate (GFR) in controls (Con) and diabetic (STZ) mice. For statistical differences an one-way ANOVA was performed in **(A)**, *n* = 9-22, and an unpaired *t*-test in **(B)**, *n* = 9-13.Values are expressed as mean ± SD. *p* < 0.05.

Treatment of non-diabetic mice with empagliflozin or enalapril/empagliflozin reduced slightly serum glucose levels compared to the control/placebo group (Figure 1A, 11.5 ± 2.4 mmol control/empagliflozin or 12.6 ± 2.3 mmol control/enalapril/empagliflozin vs. 14.9 ± 3.4 mmol control/placebo). In diabetic mice, treatment with empagliflozin numerically reduced blood glucose levels compared to the placebo group without reaching significance (Figure 1A, diabetic/empagliflozin 33.3 ± 4.6 mmol/l vs. diabetic/placebo 39.0 ± 6.6 mmol/l). In the combined treatment group of enalapril/empagliflozin, a significant blood glucose-lowering effect was noted (Figure 1A, diabetic/enalapril/empagliflozin 28.1 ± 9.2 mmol vs. diabetic/placebo 39.0 ± 6.6 mmol, *p* = 0.001). Furthermore, empagliflozin treatment induced pronounced glucosuria in non-diabetic animals (Table 1). Without empagliflozin treatment no urinary glucose was detectable in non-diabetic controls. In diabetic mice, urinary glucose levels were significantly higher than in the non-diabetic groups without differences between the treatment groups. Empagliflozin and enalapril/empagliflozin treatments increased urinary volume significantly in the control group. Similar to glucosuria, urinary volume was already increased under diabetic conditions without any impact of the different treatments. The urine osmolality was not influenced by enalapril or/and empagliflozin in the diabetic animals (Supplementary Figure S1). Measurement of serum angiotensin-converting enzyme (ACE) activity showed a significant reduction after enalapril or enalapril/empagliflozin treatment in both, the non-diabetic controls and diabetic groups.

### Hemodynamic changes in snGFR by enalapril or/and empagliflozin treatment

**FIGURE 2:**
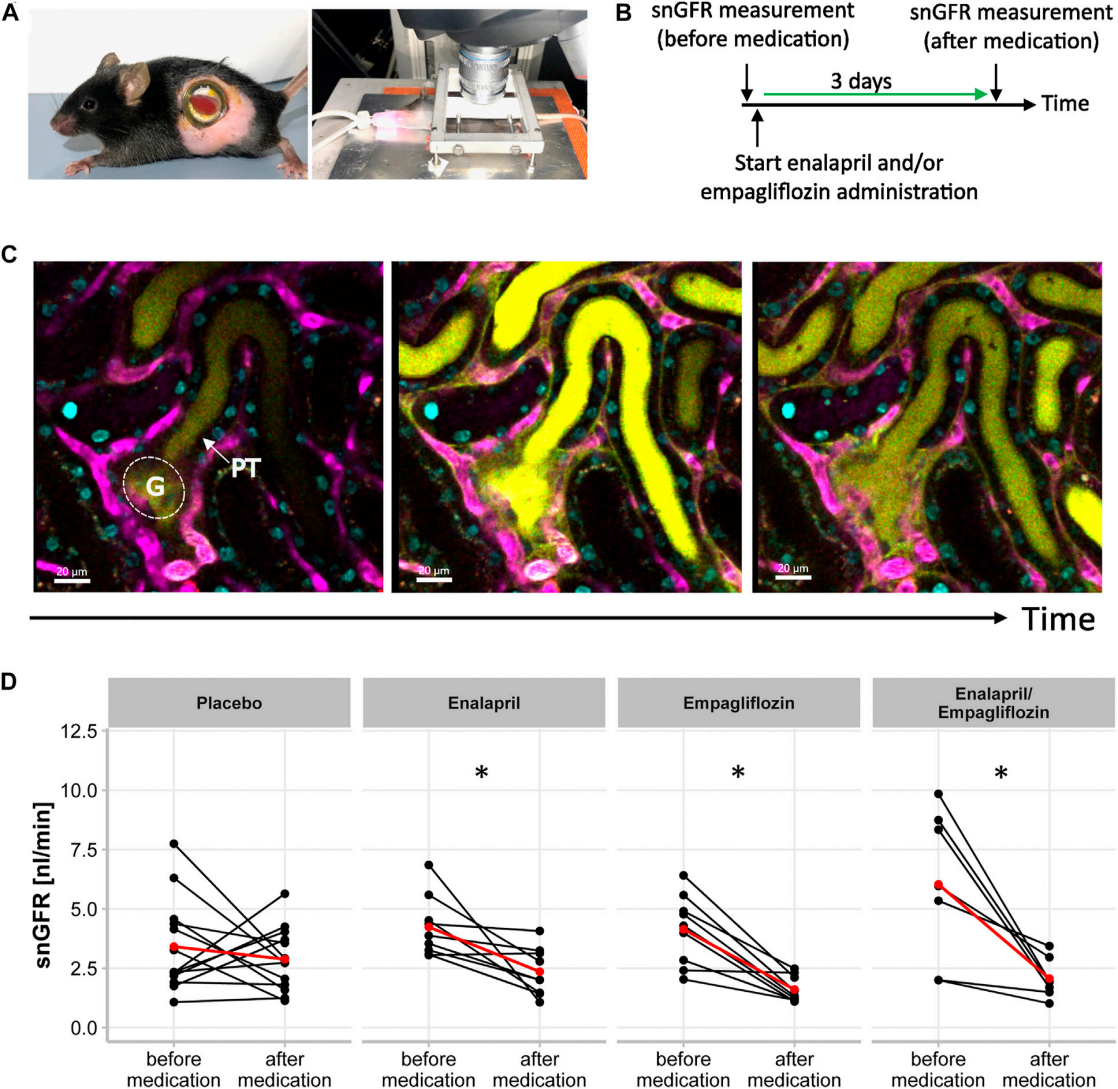
Single Nephron Glomerular Filtration Rate in STZ-diabetic mice. **(A)** Experimental setup for intravital microscopy. Mouse with an abdominal body window for repetitive imaging (right) and anesthetized mouse (endotracheal intubation) with extoriorized and stabilizied kidney for use in upright-imaging systems (left). **(B)** Timeline of snGFR measurement in the same nephrons of STZ-diabetic mice. After first snGFR measurement (before medication) administration of placebo, enalapril, empagliflozin, and enalapril/empagliflozin started. Three days later (after medication) the snGFR of the same nephron is measured again. **(C)** Beginning of filtration of the freely filtered Lucifer Yellow of one single glomerulus (G) with its subsequent proximal tubule (PT). The middle picture displays the completely filled proximal tubule and in the left picture, the filtration process is completed. The blood vessels are fluorescently labeled in magenta and the nuclei in cyan. **(D)** Alterations of snGFR in the same nephrons before and after 3 days of placebo, enalapril, empagliflozin and enalapril/empagliflozin administration in STZ-diabetic mice. Each point connected by a line represents the effect on GFR of one single nephron by the mentioned medication. The red point connected by a line indicates the mean value each before and after medication. A paired *t*-test was performed for statistical differences before and after medication in the same nephron. *p < 0.05 before vs. after medication.

First, we confirmed the expected glomerular hyperfiltration in kidneys of diabetic mice compared to non-diabetic controls by transdermal GFR measurements (Figure 1B). To analyze hemodynamic changes in glomerular filtration in response to enalapril, empagliflozin, and enalapril/empagliflozin treatment, we measured the acute effects on snGFR longitudinally within the same nephrons of diabetic mice. The experimental setup and a representative measurement is shown in Figures 2A–C and the supplementary material (Supplementary Figure S2). As expected, snGFR remained unaltered in placebo-treated diabetic mice (Figure 2D). In contrast, treatment with the ACEi enalapril reduced snGFR by ∼44% (Figure 2D; treated 2.36 ± 1.0 nl/min vs. control 4.24 ± 1.28 nl/min, *p* = 0.014). Empagliflozin treatment reduced snGFR by ∼61% (Figure 2D; treated 1.6 ± 0.55 nl/min vs. control 4.12 ± 1.5 nl/min, *p* = 0.0006). The combination therapy with enalapril and empagliflozin reduced snGFR by ∼65% (Figure 2D; treated 2.07 ± 0.84 nl/min vs. control 6.03 ± 3.17 nl/min, *p* = 0.014).

### Alterations in afferent and efferent arteriole diameter by enalapril or/and empagliflozin treatment

**FIGURE 3:**
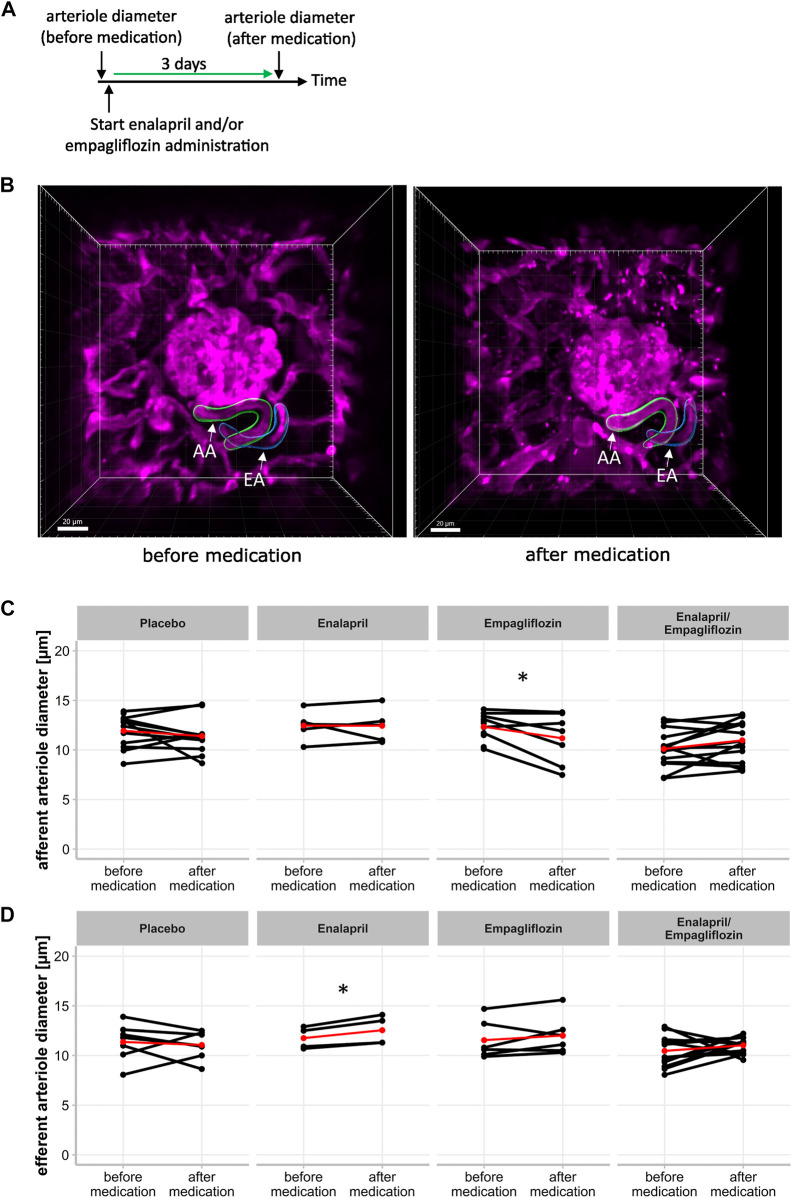
Effect of enalapril, empagliflozin and enalapril/empagliflozin on afferent and efferent arteriole alterations in STZ-diabetic mice. **(A)** Timeline of experimetal setup for afferent and efferent arteriole diameter measurement. First measurement (before medication) of arteriole diameter was obtained before the start of placebo, enalapril, empagliflozin and enalapril/empagliflozin administration. The second measurement was performed after 3 days of the respective drug treatment (after medication). **(B)** Three dimensional image of afferent (AA) and efferent (EA) arteriole of one glomerulus before medication (left) with three dimensional reconstructed afferent (green) and efferent (blue) arteriole for mean diameter calculation. The right image shows the same afferent and efferent arteriole after 3 days of either placebo, enalapril, empagliflozin, or enalapril/empagliflozin treatment (after medication). **(C)** Afferent and **(D)** efferent arteriole diameter. Same glomeruli are measured before and after respective medication. Each point connected with a line indicates the same afferent or efferent arteriole of one glomerulus on both days. The red point connected by a line indicates the mean value each before and after medication. *p < 0.05 before vs. after medication.

Beside snGFR, the width of glomerular afferent and efferent arterioles can be captured intravitally in single glomeruli and measured longitudinally via three-dimensional reconstruction. Three-dimensional images were obtained on the same days as snGFR measurements were performed (Figure 3A). The three-dimensional reconstruction of the afferent and efferent arteriole was performed afterwards (Figure 3B). Inhibition of SGLT2 with empagliflozin for 3 days led to a significant diameter reduction of the afferent arteriole (Figure 3C; treated 11.19 ± 2.55 µm vs. control 12.35 ± 1.32 µm, *p* = 0.044) in diabetic mice. Treatment with either placebo (Figure 3C; treated 11.4 ± 1.83 µm vs. control 12.09 ± 1.56 µm), enalapril (Figure 3C; treated 12.44 ± 1.70 µm vs. control 12.46 ± 1.51 µm), or enalapril/empagliflozin (Figure 3C; 10.97 ± 2.00 µm vs. control 10.42 ± 2.03 µm) had no influence on afferent arteriole width compared to the same glomeruli before treatment. In contrast, enalapril treatment led to a significant relaxation of efferent arterioles in the same glomeruli (Figure 3D; treated 12.55 ± 1.46 µm vs. control 11.92 ± 1.04 µm, *p* = 0.022). Neither placebo (Figure 3D; treated 11.06 ± 1.40 vs. control 11.18 ± 1.50), nor empagliflozin treatment alone (Figure 3D; treated 12.02 ± 1.96 µm vs. control 12.04 ± 1.79 µm), nor the combination therapy with enalapril/empagliflozin (Figure 3D; 11.03 ± 0.79 µm to 10.75 ± 1.61 µm) had an effect on the diameter of the efferent arterioles. The glomerular volume was not influenced by either enalapril or empagliflozin treatment. The combination therapy with enalapril and empagliflozin also did not change the glomerular volume (Supplementary Figure S3).

## Discussion

SGLT2i treatment reduces hyperglycemia in diabetic patients (Zinman et al., 2015) and in animal models (Vallon et al., 2013). Additionally, SGLT2i were the first class of antidiabetic drugs significantly reducing mortality, cardiovascular and renal events in diabetic patients (Wanner et al., 2016; Neal et al., 2017). Meanwhile, these beneficial effects of SGLT2i have been equally demonstrated in patients without diabetes but with cardiovascular and renal diseases suggesting a pronounced generalized mode of action that is even independent on hyperglycemia and diabetes mellitus as a disease per se (Teo et al., 2021). Considering that SGLT2 is solely expressed within the proximal tubules (Chen et al., 2010), overall- and nephroprotective effects in humans are likely to be mediated via the kidneys, but the exact mechanisms and the role of hemodynamic effects especially in combination therapy with ACEi/ARBs are controversially discussed. While RAS inhibition was the standard nephroprotective therapy in DN for many years, in recent studies SGLT2i were beneficial despite 80% or more of all patients that were already on ACEi/ARB medication (Zinman et al., 2015; Wanner et al., 2016). Recent experimental studies even showed that the simultaneous administration prevented the development of renal injury more than SGLT2i or ACEi alone (Kojima et al., 2015). To compare the hemodynamic mechanisms of single and combined ACEi/SGLT2i therapy, we measured snGFR and arteriole width regulation in response to enalapril, empagliflozin, and enalapril/empagliflozin administration in the same nephrons on different days via longitudinal intravital microscopy.

Enalapril treatment significantly reduced snGFR in the diabetic group compared to placebo treated animals. The snGFR reduction by enalapril was accompanied and likely caused by efferent arteriole dilation, while no effect was seen regarding width regulation of the afferent arteriole. This interpretation of our findings fits the paradigm that the angiotensin type 1 receptors are expressed stronger in the efferent arteriole than in the afferent arteriole. (Hall et al., 1977; Schrankl et al., 2021). Another intravital microscopy study in STZ-diabetic rats showed restored arteriole width regulation and diminished hyperfiltration in response to ARBs (Satoh et al., 2010). In diabetic patients, hypertension is a relevant comorbidity. ACEi/ARBs are effective drugs to regulate hypertension by efferent vasodilation with a fall in filtration pressure. Long term clinical trials showed slower renal function loss in CDK and lower cardiovascular mortality (Perico, Ruggenenti and Remuzzi, 2017).

In contrast to enalapril, successful snGFR reduction by SGLT2i seems to be promoted by afferent arteriole vasoconstriction but not via width regulation of the efferent arteriole as supported by our *in vivo* study results in type 1 diabetic mice. SGLT2i are shown to activate the production of the TGF mediator adenosine, causing vasoconstriction of the afferent arteriole by binding to the adenosine A1 receptor (Alicic, Johnson and Tuttle, 2018). In humans little is known about the correlation of TGF, adenosine and SGLT2 inhibition. Patients with type 1 diabetes showed increased urinary adenosine production in response to empagliflozin treatment, suggesting that reduced GFR and afferent arteriole constriction was related to increased adenosine production by *macula densa* (Rajasekeran et al., 2017). Kidokoro et al. (2019) showed afferent arteriole vasoconstriction in type 1 diabetes Akita mice in response to SGLT2i *in vivo,* which was abolished by A1 adenosine receptor antagonist. This data further promotes the importance of the adenosine signaling pathway for TGF regulation (Kidokoro et al., 2019). The opposite was shown in patients with type 2 diabetes, here preliminary data supported that SGLT2 inhibition by dapagliflozin may also lower GFR by vasodilation of the efferent arteriole (van Bommel et al., 2020). These controversial results of SGLT2 inhibition in type 1 versus type 2 diabetes models needs further investigation to clarify the arteriole tone regulation in response to SGLT2i *in vivo*.

The combination therapy of ACEi/SGLT2i did reduce snGFR equally to SGLT2i alone. Unexpectedly, in the combined ACEi/SGLT2i therapy approach, snGFR was lowered without any obvious afferent or efferent arteriole width regulation. Currently, we cannot provide a clear explanation for this finding. The reduction of snGFR was not accompanied by osmotic changes in the urine. The snGFR changes were also not related to changes in the glomerular volume most probably because of the short period of treatment. Interestingly, simultaneous administration of both ACEi/SGLT2i drugs does not synergistically reduce intraglomerular pressure as with ACEi and NSAIDs (Lapi et al., 2013; Joannidis and Hoste, 2018). In this context, combination therapy of ACEi with NSAIDs but not together with SGLT2i increases the risk of acute renal failure suggesting a specific interacting or novel regulatory mechanism preserving renal function with combined ACEi/SGLT2i therapy. Hereby, adenosine, as a TGF mediator and angiotensin II, the main effector of the RAS are known to interact to regulate glomerular hemodynamics. Low concentration of the afferent arteriole vasoconstrictor adenosine increases the response to angiotensin II on the efferent arteriole. In contrast, physiological concentration of angiotensin II increases the contractility as response to adenosine. Moreover, the addition of nitric oxide abolished both modulating effects regulating TGF responses (Persson et al., 2013). Additional investigations are needed to clarify the involved mechanisms of a combined therapy in detail. Besides hemodynamic regulation, alternative renoprotective mechanisms may become increasingly important especially under combination therapy. SGLT2i also inhibits proximal tubular reabsorption reducing active tubular transport work. This may redistribute energy demand and oxygen consumption in the kidney, thereby upregulating erythropoietin production with potential protective effects (Nespoux and Vallon, 2020). We and others showed that SGLT2i ameliorated also inflammation and mesangial matrix expansion in a mouse model of DN. Future studies need to investigate, whether RASi or SGLT2i may regulate juxtaglomerular (next to the afferent arteriole) renin-lineage cell phenotype and recruitment as a well described regenerative response to injury mechanism besides the known classical hemodynamic TGF pathway connection (Sequeira López et al., 2004).

Overall, it also needs to be considered that slight differences exist between SGLT2 regulation in mice and humans. While SGLT2 seems to be usually upregulated in diabetic kidney disease in man, in our STZ-induced kidney model as well as in many other experimental diabetic disease models renal SGLT2 expression is not upregulated which limits the SGLT2i-mediated increase in urinary glucose levels in diabetic mice (Albertoni Borghese et al., 2009; Vallon et al., 2013).

Despite the strength of the *in vivo* visualization of direct effects on renal hemodynamics, our study has also limitations. We cannot exclude any dose-dependent effects of ACEi and/or SGLT2i, especially in the combined treatment group. Furthermore, the direct effect of ACEi and/or SGLT2i was only measured shortly after start of treatment within the same nephron. Combined therapy with ACEi and SGLT2i showed better outcomes in clinical trials compared to each treatment alone (Kojima et al., 2015). Visualization of single glomeruli with a subsequent proximal tubule in mice with the used age is very challenging. Next to the limited number of glomeruli suitable for measuring snGFR and arteriole width, the visualization of nephrons deeper than 100 µm is technically not possible. Therefore, all studies concerning snGFR and arteriole width regulation were performed on superficial glomeruli. This, together with the general functional heterogeneity within the glomeruli represents a major limiting factor of our study. Further investigation of long-term effects regarding snGFR and arteriole width would help to understand the renoprotective effects in more detail.

In conclusion, we directly visualized the hemodynamic actions of RAS or SGLT2 inhibition on single glomeruli in type 1 diabetic mice. Therein, snGFR was reduced via either efferent arteriole vasodilation (ACEi) or afferent arteriole vasoconstriction (SGLT2i), which might be responsible for long term renoprotective effects. The decreased snGFR without changes in arteriole width in diabetic mice with combined ACEi and SGLT2i therapy suggests unknown interactions of both drug groups potentially at the level of *macula densa* cell regulation as well as additional renoprotective mechanisms.

## Data Availability

The original contributions presented in the study are included in the article/supplementary material, further inquiries can be directed to the corresponding author.
