# Supplementary figures and images for: Intravital imaging of hemodynamic glomerular effects of enalapril or/and empagliflozin in STZ-diabetic mice
Source: Front Physiol. 2022 Sep 12;13:982722. doi: 10.3389/fphys.2022.982722 (PMC9511053; doi:10.3389/fphys.2022.982722)

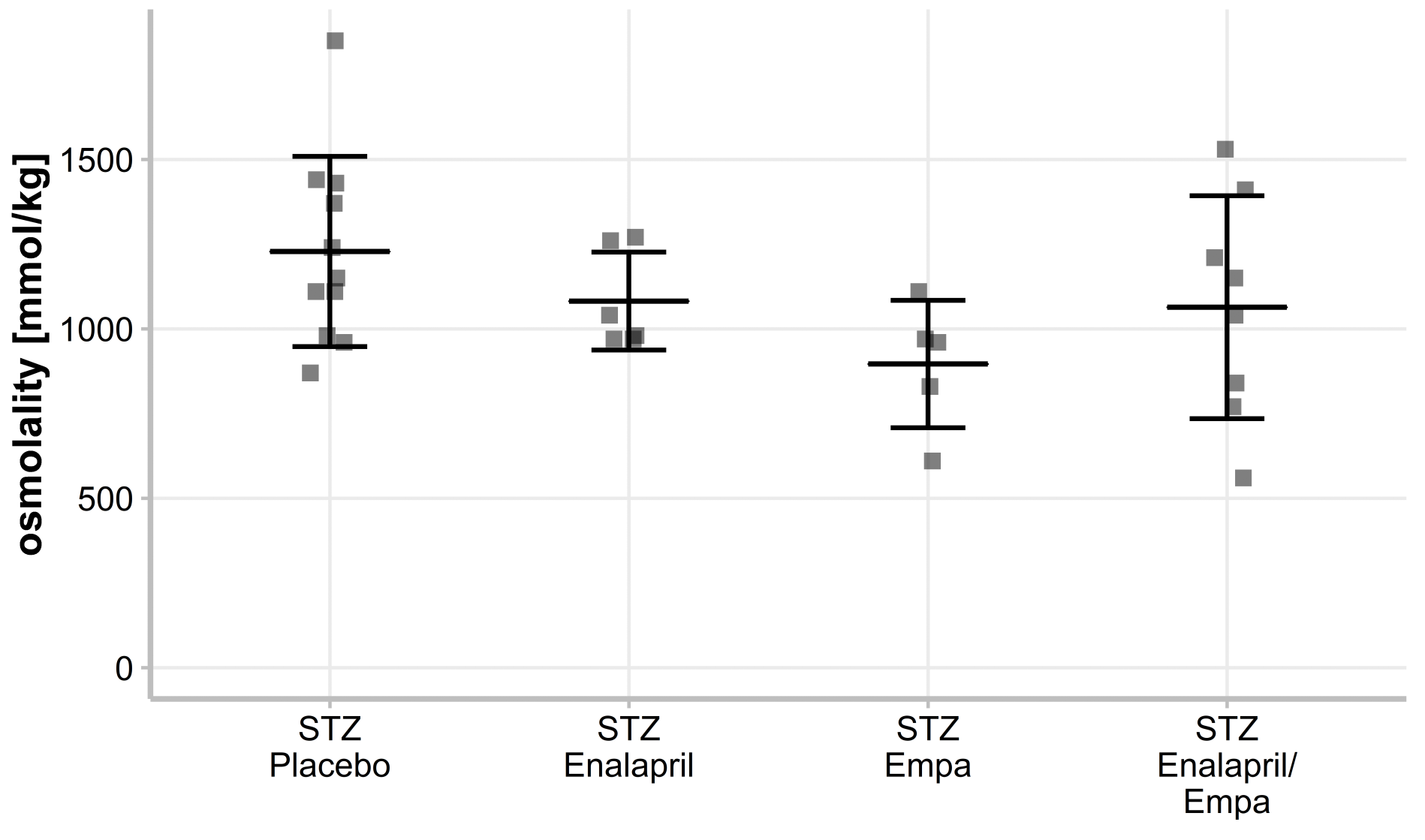

Supplement: Supplementary file 1 [file Image1.TIFF]

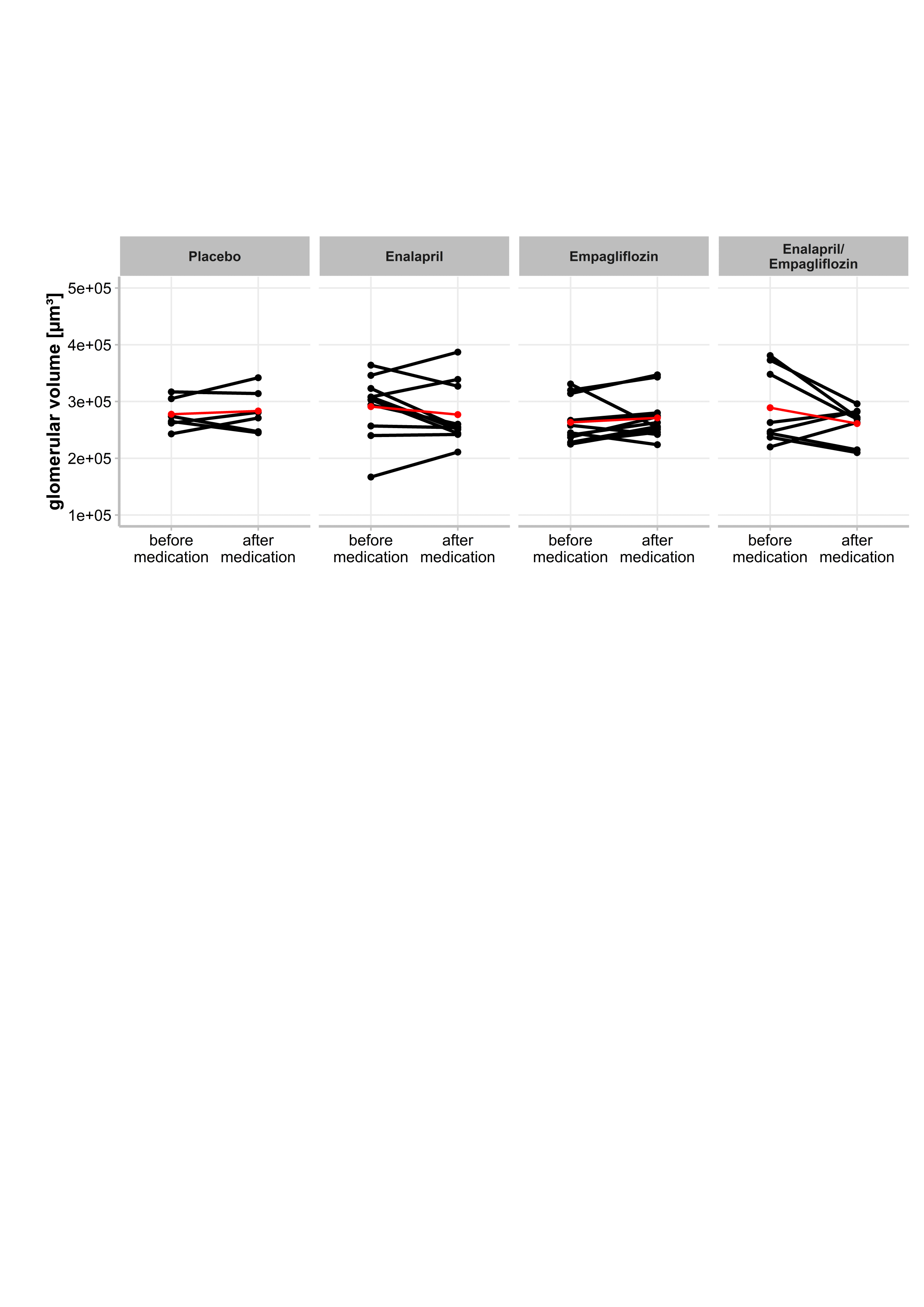

Supplement: Supplementary file 3 [file Image2.TIFF]
